# Efficacy and Safety of Single and Double Doses of Ivermectin versus 7-Day High Dose Albendazole for Chronic Strongyloidiasis

**DOI:** 10.1371/journal.pntd.0001044

**Published:** 2011-05-10

**Authors:** Yupin Suputtamongkol, Nalinee Premasathian, Kid Bhumimuang, Duangdao Waywa, Surasak Nilganuwong, Ekkapun Karuphong, Thanomsak Anekthananon, Darawan Wanachiwanawin, Saowaluk Silpasakorn

**Affiliations:** 1 Department of Medicine, Faculty of Medicine Siriraj Hospital, Mahidol University, Bangkok, Thailand; 2 Department of Preventive and Social Medicine, Faculty of Medicine Siriraj Hospital, Mahidol University, Bangkok, Thailand; 3 Department of Parasitology, Faculty of Medicine Siriraj Hospital, Mahidol University, Bangkok, Thailand; Texas Tech University Health Sciences Center, United States of America

## Abstract

**Background:**

Strongyloidiasis, caused by an intestinal helminth *Strongyloides stercoralis*, is common throughout the tropics. It remains an important health problem due to autoinfection, which may result in hyperinfection and disseminated infection in immunosuppressed patients, especially patients receiving chemotherapy or corticosteroid treatment. Ivermectin and albendazole are effective against strongyloidiasis. However, the efficacy and the most effective dosing regimen are to be determined.

**Methods:**

A prospective, randomized, open study was conducted in which a 7-day course of oral albendazole 800 mg daily was compared with a single dose (200 microgram/kilogram body weight), or double doses, given 2 weeks apart, of ivermectin in Thai patients with chronic strongyloidiasis. Patients were followed-up with 2 weeks after initiation of treatment, then 1 month, 3 months, 6 months, 9 months, and 1 year after treatment. Combination of direct microscopic examination of fecal smear, formol-ether concentration method, and modified Koga agar plate culture were used to detect strongyloides larvae in two consecutive fecal samples in each follow-up visit. The primary endpoint was clearance of strongyloides larvae from feces after treatment and at one year follow-up.

**Results:**

Ninety patients were included in the analysis (30, 31 and 29 patients in albendazole, single dose, and double doses ivermectin group, respectively). All except one patient in this study had at least one concomitant disease. Diabetes mellitus, systemic lupus erythrematosus, nephrotic syndrome, hematologic malignancy, solid tumor and human immunodeficiency virus infection were common concomitant diseases in these patients. The median (range) duration of follow-up were 19 (2–76) weeks in albendazole group, 39 (2–74) weeks in single dose ivermectin group, and 26 (2–74) weeks in double doses ivermectin group. Parasitological cure rate were 63.3%, 96.8% and 93.1% in albendazole, single dose oral ivermectin, and double doses of oral ivermectin respectively (P = 0.006) in modified intention to treat analysis. No serious adverse event associated with treatment was found in any of the groups.

**Conclusion/Significance:**

This study confirms that both a single, and a double dose of oral ivermectin taken two weeks apart, is more effective than a 7-day course of high dose albendazole for patients with chronic infection due to *S. stercoralis*. Double dose of ivermectin, taken two weeks apart, might be more effective than a single dose in patients with concomitant illness.

**Trial Registration:**

ClinicalTrials.gov NCT00765024

## Author Summary

Strongyloidiasis, caused by an intestinal helminth *Strongyloides stercoralis*, is common throughout the tropics. We conducted a prospective, clinical study to compare the efficacy and safety of a 7-day course of oral albendazole with a single dose of oral ivermectin, or double doses, given 2 weeks apart, of ivermectin in Thai patients who developed this infection. Patients were regularly followed-up after initiation of treatment, until one year after treatment. Ninety patients were studied (30, 31 and 29 patients in albendazole, single dose, and double doses ivermectin group, respectively). The average duration of follow-up were 19 (range 2–76) weeks in albendazole group, 39 ( range 2–74) weeks in single dose ivermectin group, and 26 ( range 2–74) weeks in double doses ivermectin group. Parasitological cure rate were 63.3%, 96.8% and 93.1% in albendazole, single dose oral ivermectin, and double doses of oral ivermectin respectively. No serious adverse event associated with treatment was found in any of the groups. Therefore this study confirms that both a single, and a double dose of oral ivermectin taken two weeks apart, is more effective than a 7-day course of high dose albendazole for patients with chronic infection due to *S. stercoralis*.

## Introduction

Infection with the intestinal helminth *Strongyloides stercoralis* remains a common problem throughout the tropics, including Thailand [1], [2]. It is estimated that 30 to 100 million people are infected worldwide [1]. Most infected individuals are asymptomatic or developed minimally symptomatic chronic infection through autoinfection [3]. Potentially fatal disseminated infections, due to an acceleration of the autoinfection cycle, are seen in immunocompromised patients, such as those with concurrent human T-lymphotropic virus-1 (HTLV-1) infection, or those on corticosteroid therapy [3], [4]. Other recognized risk factors for disseminated strongyloidiasis include malignancies especially lymphoma, organ transplantation and diabetes mellitus [5], [6]. Gastrointestinal symptoms associated with strongyloidiasis include diarrhea, abdominal discomfort, nausea/vomiting and anorexia. The diagnosis of strongyloidiasis should be suspected if there are clinical signs and symptoms, or eosinophilia [7]. Definitive diagnosis of strongyloidiasis is usually made on the basis of detection of larvae in the stool [8]. The combination of diagnostic approaches such as repeated direct microscopic examination of fecal smear, fecal concentration methods such as formol-ether concentration (FEC), and modified Koga agar plate culture have been used to improve the likelihood of detecting this parasite [7]–[11].

In the past, the treatment of choice for strongyloidiasis has been thiabendazole, but this drug has unpleasant side effects and is no longer available. Albendazole, another broad-spectrum antihelmintic agent, was previously shown to be effective against *S. stercoralis* [12]–[15]. More recent reports suggest ivermectin, a macrolide-like agent developed primarily for the treatment of onchocerciasis, is as effective as thiabendazole [16] and superior to albendazole against intestinal strongyloidiasis [17]–[21].

Although a single dose of ivermectin 200 microgram/kilogram body weight (µg/kg) was shown to be effective in uncomplicated chronic strongyloidiasis, repeated treatment at two or three week intervals was thought to be necessary to eliminate larvae generated by autoinfection [22].

A preparation of oral ivermectin licensed for human use has recently become available in Thailand. However, albendazole remains the most widely used antiparasitic drugs for the treatment of this infection in this country. The purpose of the present study was to assess the safety and efficacy of a single dose of ivermectin (200 µg/kg), or two doses of ivermectin given 2 weeks apart, and a 7-day course of high dose albendazole for the treatment of chronic strongyloidiasis in adult patients who were at high risk of hyperinfection or disseminated infection.

## Materials and Methods

### Study design and ethics

This was a prospective open-label, randomised, controlled study conducted between July 2008 and April 2010 at Siriraj Hospital, Faculty of Medicine Siriraj Hospital, Mahidol University, Bangkok, Thailand. The study was approved by the Ethical Committee on Research Involving Human Subjects, Siriraj Hospital, Faculty of Medicine, Mahidol University, Thailand. All patients were informed about the purpose of the trial and gave written informed consent before enrollment. The study enrollment was stopped in December 2009 after 100 eligible patients had been recruited.

### Patients

Adult patients (>18 years) were recruited from Siriraj Hospital if characteristic rhabditiform larvae of *S. stercoralis* were present on fecal microscopy. Exclusion criteria included a history of allergic reaction to either study medication, treatment within the month prior to the study with any drug known to have anti-strongyloides activity, pregnancy or lactation and any suggestion of disseminated strongyloidiasis.

### Treatment

Computer generated, simple, random allocation sequences were prepared for 3 study groups by the investigator team. These were sealed in an opaque envelope and numbered. The investigator (YS) assigned study participants to their respective treatment group after opening the sealed envelope. Once an eligible patient was identified and informed consent was obtained, the patient was randomly allocated to one of the following group (1∶1∶1 ratio):

1. Ivermectin-I group: a single oral dose of 200 µg/kg (Vermectin®, Atlantic Laboratories Co, Ltd., Thailand).
2. Ivermectin-II group: two oral doses of 200 µg/kg of ivermectin (Vermectin®, Atlantic Laboratories Co, Ltd., Thailand) given 2 weeks apart.
3. Albendazole group: oral albendazole (Albatel®, TO Chemical, Thailand) 400 mg twice daily for 7 days.

### Study Procedures

Baseline evaluation included history, detailed physical examination, and laboratory investigations such as complete blood count (CBC), urinalysis, and biochemistry. Patients were requested to collect two consecutive fecal samples at every hospital visit. The coprodiagnosis for the detection of *S. stercoralis* larvae using direct smear, formol-ether concentration method [9], and modified Koga agar plate culture method [10] was performed for each patient at the Infectious Diseases and Tropical Medicine Laboratory, Division of Infectious Diseases and Tropical Medicine, Faculty of Medicine Siriraj Hospital, Mahidol University, Thailand.

Patients were required to make seven hospital visits to complete the study: at baseline evaluation and initiation of treatment, at 2 weeks after initiation of treatment, then at 1 month, 3 months, 6 months, 9 months, and 1 year after treatment. Patients who completed 1 year of follow-up were invited for further follow-up visits every 3 months or at their convenience.

### Outcome measures

#### Efficacy

The efficacy of treatment was analyzed on a modified intention to treat, and a per-protocol basis. Modified intention to treat analysis was based on the number of patients who were randomized and received treatment. Per-protocol analysis was based on the number of patients who completed the treatment and were followed up as planned. Analyses of adverse events were on the modified intention to treat basis.

The outcome was evaluated according to the following definitions; **“Cure”** was defined as clinical improvement (if symptomatic before treatment) and the absence of rhabditiform larvae in the stool at day14 of treatment and throughout the follow-up period. “**Failure**” was defined as the presence of larvae two weeks after initiation of treatment or the reappearance of larvae during follow-up.

“Adverse events” were defined as symptoms or signs that developed after the study drug administration and had not been reported prior to the administration of the first dose of the antihelmintic.

#### Sample Size and Statistical Methods

Assuming the therapeutic efficacy of albendazole to be 60% and of both regimens of ivermectin treatment to be 90% [17]–[20], [22], with alpha error 0.5, and 80% power, it was calculated that 22 patients would be needed in each study group. The lost to follow up rate was assumed to be 20%. Therefore 27 patients would be needed in each study group.

Demographic data, results of investigations, stool examination at baseline and follow up visits were recorded in the database. All statistical analyses were performed using SPSS, version 17.5. Pearson chi square or Fisher's exact tests were used to compare rates and proportions, as appropriate. Mann-Whitney U tests were used to analyze continuous variables that were not normally distributed. Independent sample t- tests were used to compare normally distributed variables, taking a probability of less than 5% as the level of significance. Kaplan-Meier plot and Cox proportional hazard model were constructed to identify independent risk factors for treatment failure.

## Results

**Figure 1:**
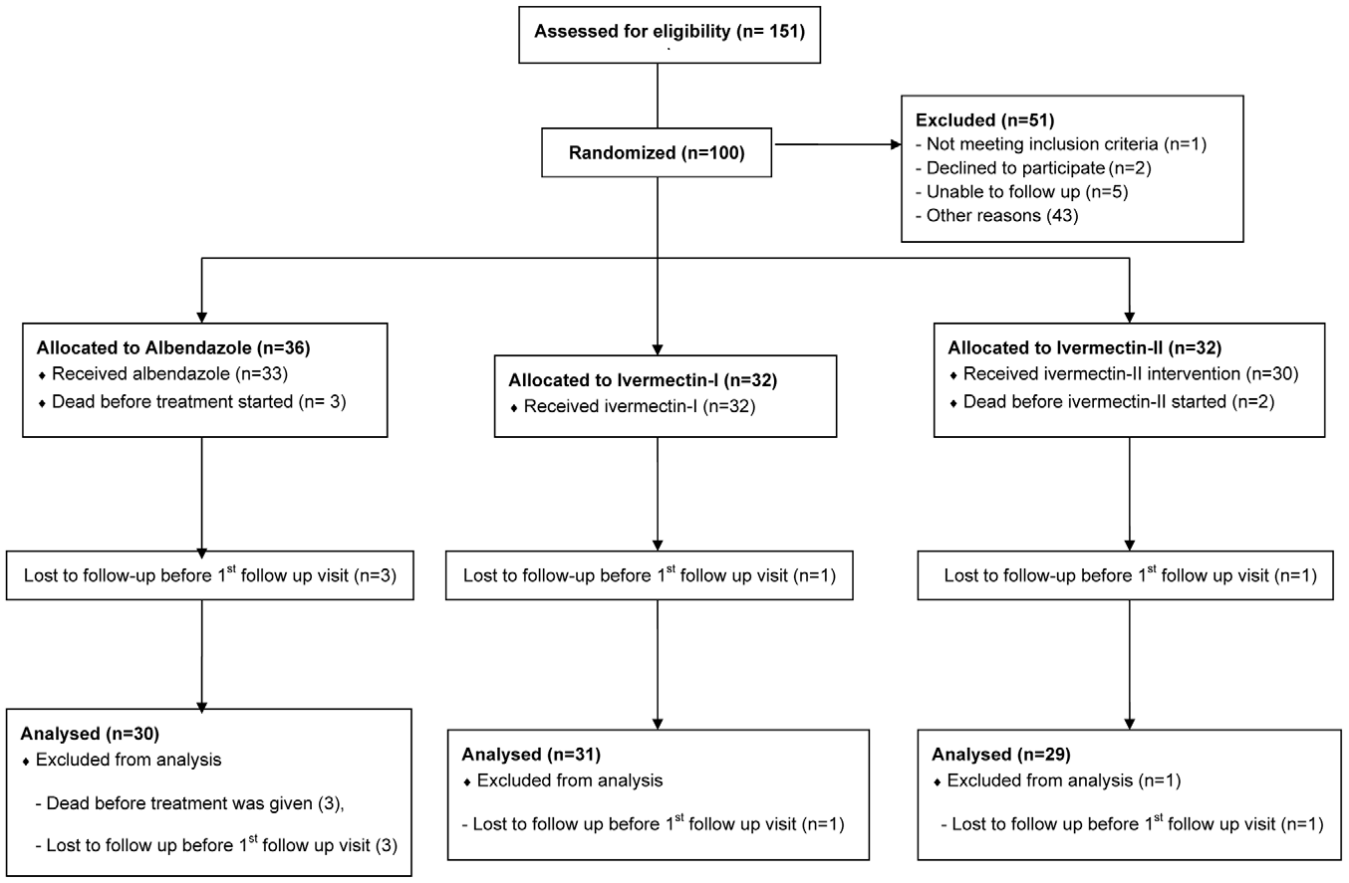
Total number of enrollment, randomization, follow-up, and inclusion in the final analysis comparing among three treatment groups.

One hundred and fifty one patients had detectable rhabditiform larvae of *S. stercoralis* on fecal microscopy during the study period. One hundred patients were enrolled (36, 32, and 32 patients in albendazole, ivermectin-I, and ivermectin-II groups respectively). Ten patients were excluded from analysis because they did not receive or complete the study treatment (3 in albendazole group, 2 in ivermectin-II group), or they were lost to follow-up immediately after treatment (3 in albendazole group, 1 each in ivermectin-I and ivermectin-II respectively). Overall, 90 patients were eligible for the modified intention to treat analysis. Detail of the total number of enrollment, randomization, follow-up and inclusion in the final analysis comparing among the three treatment groups is shown in Figure 1.

The demographic data, concomitant diseases, baseline clinical and laboratory investigations are shown in Table 1 and 2. All except one patient had an associated medical problem, including concurrent other parasitoses. These patients also had abnormal serum aspartate aminotranferase (AST) and alanine aminotransferase (ALT) levels prior to entering the study due to their underlying conditions.

**Table 1:** Demographic and baseline clinical features of the three study groups.

Characteristics	Albendazol(N = 30)	Ivermectin-I(N = 31)	Ivermectin –II(N = 29)	P-value
Male: female	21∶9	21∶10	19∶10	0.934
Median (range) age, yr	54 (23–81)	51 (29–77)	52 (25–78)	0.273
Median (range) weight, kg	59 (37–80)	57 (37–85)	59 (44–73)	0.75
Concomitant illnesses, n (%)				0.607
- None	1	0	0	
- Diabetes mellitus	8	6	5	
- NS/SLE	4	5	4	
- AIDS/HIV infection	3	2	5	
- Hematological malignancy	2	3	4	
- Solid tumor	3	1	6	
- Rheumatologic diseases	2	1	1	
- Chronic kidney disease	3	2	2	
- Alcohol drinker	3	1	1	
- Others	6	12	4	
Immunosuppressive drug, n (%)	10 (33.3)	11(35.5)	11 (37.9)	0.934
Concomitant parasitoses*				0.380
- Hookworm infection	2	1	0	
- Opisthorchiasis	2	1	2	
- Enterobious infection	0	1	0	
- *Entamoeba histolytica* infection	0	1	0	
- *Isospora belli* infection	0	0	1	

*Diagnosis obtained by ova or cyst found from fecal examination.

**Table 2:** Comparison of symptoms related to chronic strongyloidiasis and baseline laboratory results.

Parameters	Albendazol(N = 30)	Ivermectin-I(N = 31)	Ivermectin –II(N = 29)	P-value
Symptoms associated with strongyloidiasis, n (%)				
- Diarrhea	14 (46.7)	11 (35.5)	16 (51.6)	0.609
- Abdominal pain	3 (10)	4 (12.9)	6 (20.7)	0.483
- Nausea/vomiting	4 (13.3)	4 (12.9)	6 (20.7)	0.650
Laboratory test, mean (SD)				
- Hematocrit, % (35–45)	32.7 (7)	35.4 (6)	32 (8)	0.132
- Eosinophil count, ×10^6^/L (<500)	967(1,239)	1,203(2,714)	554(1,781)	0.366
- Total eosinophil >500/µL, n (%)	14 (46.7)	18 (58.1)	13 (44.8)	0.535
- AST, U/L (0–37)	45(43)	45(60)	38(34)	0.805
- ALT, U/L (0–40)	38(31)	42(38)	38(47)	0.924
- Creatinine, mg/dL (0.8–1.2)	1.2(0.5)	1.1(0.9)	0.8(0.8)	0.933

The intensity of initial infection of the three study groups was similar, i.e. *S. stercoralis* larvae were found from the direct fecal examination in 24 (80%), 25 (80.6%), and 28 (96.6%) in albendazole, Ivermectin-I, and ivermectin–II groups, respectively (P = 0.123). Larvae were also detected from modified Koga agar plate culture in 22/26 (84.6%) patients in the albendazole group, in 22/26 (84.6%) patients in the ivermectin-I group, and in 24/29 (82.8%) patients in the ivermectin-II group, respectively (P = 0.976).

Diarrhea was detected in half of the patients and it was relieved after treatment in most patients. Abnormal bowel movement at second week of follow-up was reported in 4 patients in the albendazole group, 2 patients in the ivermectin-I group, and 3 patients in the ivermectin-II group, respectively (P = 0.641). *S. stercoralis* larvae were detected in one patient in the ivermectin-I group at third month of follow-up. In ivermectin-II group, *S. stercoralis* larvae were detected at second week prior to the second dose of ivermectin in two patients. No patients had reinfection/relapse after the second dose of ivermectin treatment. In albendazole treated patients, *S. stercoralis* larvae were detected at second week of follow-up in 2 patients, at first month of follow-up in 2 patients, between 3–6 months of follow-up in 3 patients, and between 6–12 months of follow-up in 4 patients. All of the relapses/ reinfections found during follow-up were clinically inapparent.

**Figure 2:**
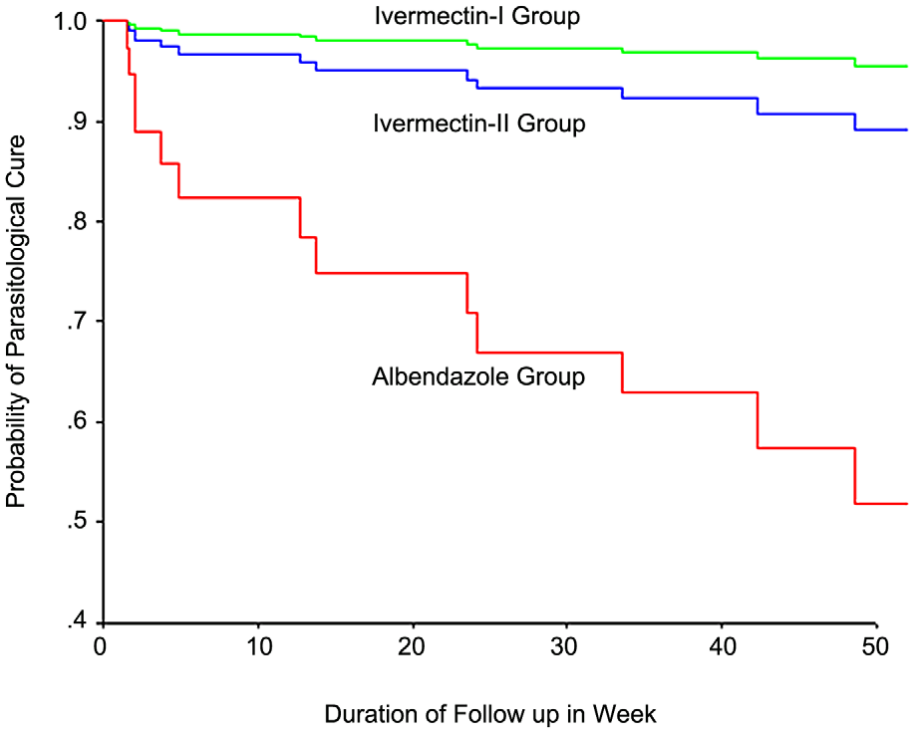
Kaplan-Meier Plot comparing the parasitological cure among the albendazole, ivermectin-I, and ivermectin-II treatment groups over one year follow-up period.

Parasitologically, parasite elimination was documented in 19 (63.3%) albendazole treated patients, in 30 (96.8%) single-dose ivermectin treated patients, and 27 (93.1%) two-dose ivermectin treated patients (P = 0.006) (Table 3). Cox regression analysis showed that albendazole treated group had 14.7 times (95%CI 1.8–111.9), and 5.7 times (95%CI 1.3–25.7) higher risk for reinfection/ relapse of strongyloidiasis than ivermectin-I and ivermectin-II group, respectively. Kaplan- Meier Plot compares the parasitological cure rate between these study groups is shown in figure 2. No hyperinfection syndrome or disseminated infection was found among these patients during the study period. *S. stercoralis* larvae were detected after treatment using FEC in 8 patients, and by modified Koga agar plate culture only in 6 of them. All patients with relapse/reinfection were retreated with two doses of ivermectin in two weeks apart.

**Table 3:** Outcome of treatment among the three study groups.

Parameters	Albendazol(N = 30)	Ivermectin-I(N = 31)	Ivermectin-II(N = 29)	P-value
Duration of follow up				
- Median (range), weeks	19(2–76)	39(2–74)	26 (2–74)	0.248
Outcome: Parasitological responses				0.006
- Elimination, n (%)	19 (63.3)	30 (96.8)	27 (93.1)	
- Failure	11 (36.7)	1 (3.2)	2 (6.9)	
- Persistence at 2 week	2	0	2	
- Relapse /reinfection	9	1	0	

Overall albendazole and ivermectin were well tolerated. Transient elevation of AST, and ALT levels was detected in one patient in ivermectin-II group. The AST and ALT levels returned to normal 2 weeks after the second dose of ivermectin treatment. Severe nausea and vomiting was reported in one patient in the albendazole group.

Fifteen patients died after enrollment (5 patients in each treatment group). Causes of death were not related to the study drugs, and were considered to be due to an underlying disease or its complications (solid tumor in 5, hematologic malignancies in 3, diabetes mellitus, or systemic lupus erythrematosus (SLE), or hypertension with complications such as myocardial infarction or sepsis in 7 patients). The median duration from enrollment to death was 2 weeks (range 2–14 weeks) in the albendazole group, 5 weeks (range 2–38 weeks) in the ivermectin-I group, and 2 weeks (range 1–27 weeks) in the ivermectin-II group, respectively.

## Discussion

Strongyloidiasis remains a significant health problem in many developing countries, mainly due to the potential for lethal disseminated disease [1], [5]. Gastrointestinal symptoms associated with strongyloidiasis found in this study included diarrhea, abdominal discomfort, nausea/vomiting and anorexia. Chronic infection with *S. stercoralis* was clinically inapparent in half of the patients at enrollment, and in all of relapses/ reinfections found during follow up. Peripheral eosinophilia (>500 eosinophils/µL.) was detected in half of the patients at enrollment. *S. stercoralis* larvae were detected after treatment using FEC in 8 patients, and by modified Koga agar plate culture in 6 patients. This information confirmed that fecal examination, including culture and/or serology, every 3–6 months of follow-up should be recommended for early detection and treatment of latent infection to prevent hyperinfection or disseminated disease in these patients [3], [5].

Results of this study corroborate the results from previous randomised controlled studies on the higher efficacy of ivermectin compared to various dosage regimens of albendazole for treating chronic strongyloidiasis [17]–[21]. A summary of results from these previous controlled trials of ivermectin treatment for chronic strongyloidiasis is shown in Table 4. Although these studies were conducted in different geographical areas and population groups, i.e. in children and adults, they were considered to be within a community-based setting, such as schools or primary care clinic. The duration of follow-up varied from 3 weeks to 12 months. The present study was conducted in a tertiary hospital. The majority of patients had known risk factors for disseminated strongyloidiasis, and approximately one-third of them received corticosteroid or chemotherapy. Results of this study confirmed that ivermectin was also effective in this population who were at high risk of severe infection.

**Table 4:** Summary of published controlled trials of oral ivermectin treatment for chronic strongyloidiasis.

Comparative Drug, Dosage Regimen	Durationof follow-up	N	Cure,N (%)	Author,year, [Ref]
1. Albendazole 400 mg/d - 3days2. Ivermectin 150–200 µg/kg, single dose	30 days	2429	9 (38)24 (83)	Datry A,1994 [17]
1. Thiabendazole 50 mg/kg/day - 3 days2. Ivermectin 200 µg/kg, single dose3. Ivermectin 200 µg/kg, - 2 days	7 days, then1, 3, 6months	191618	18(94.7)16(100)18(100)	Gann PH,1994 [16]
1. Albendazole 400 mg/d -3 days2. Ivermectin 200 µg/kg, single dose	3 weeks	149152	67(45)126(82.9)	Marti H,1996 [18]
1. Pyrvinium pamoate 5 mg/kg/d -3 days2. Albendazole 400 mg/d - 3days3. Ivermectin 6 mg 2 doses- 2 weeks apart	2 weeks,then 6, 12months	608467	14 (23.3)65 (77.4)65 (97)	Toma H,2000 [19]
1. Albendazole 400 mg/d - 5 days2. Ivermectin 150–200 µg/kg, single dose	30 days	3378	26(78.8)77(98.7)	Nontasut P,2005 [20]

Albendazole remains an option of treatment for chronic strongyloidiasis in many countries in South East Asia, where oral ivermectin is not widely available. Cure rates of a regimen consisting of albendazole 400 mg daily for three to five days varied from 38–87% in those without underlying diseases [14], [17]–[20]. In this study, the cure rate was found to be 63% when a 7-day course of high dose albendazole was used. The efficacy of albendazole varied widely even when the same dose and duration of treatment was used. Differences in duration of follow-up examinations could be one explanation, and re-infection from the environment may also be a factor when the efficacy is monitored for an extended period in endemic areas. The study which reported the highest cure rate (87%) was conducted in Okinawa, Japan [19], where the chance of re-infection from the environment was less likely to occur compared to other studies conducted in endemic areas [17], [18], [20], [21].

Two patients in the ivermectin-II group had detectable *S. stercoralis* larvae in the second week prior to the second dose of ivermectin treatment. One patient in ivermectin-I group also had detectable *S. stercoralis* larvae 3 months after treatment. This observation supports the recommendation that repeated doses of ivermectin should be the preferred treatment in patients with chronic strongyloidiasis who have an underlying or concomitant illness [22].

The limitation of this study was the high loss to follow-up rates over time. High mortality associated with the concomitant illnesses was an unavoidable cause of concern in this study. The median duration of follow up was 19 weeks in albendazole group, 39 weeks in ivermectin-I, and 26 weeks in ivermectin-II group. The non-significant shorter duration of follow up found in albendazole treatment group was due to the significant higher rate of treatment failure compared to ivermectin. However, the study still had sufficient power to detect a difference between albendazole and ivermectin treatments. This study, however, was too small to detect any but the most severe and common side- effects of both albendazole and ivermectin. Only one of albendazole treated patients and one treated with ivermectin had transient changes in transaminases, a well-recognized and reversible adverse event.

In conclusion, this clinical study confirms that both a single and a double dose of oral ivermectin taken at a two-week interval is more effective than a 7-day course of high dose of albendazole for patients with chronic infection due to *S. stercoralis*.

## Supporting Information

Checklist S1 Consort Checklist.
(0.23 MB DOC)

Protocol S1 Trial Protocol: downloaded from http://clinicaltrials.gov/ct2/show/NCT00765024.
(0.06 MB DOC)
