# Supplementary material for: Efficacy and Safety of Single and Double Doses of Ivermectin versus 7-Day High Dose Albendazole for Chronic Strongyloidiasis
Source: PLoS Negl Trop Dis. 2011 May 10;5(5):e1044. doi: 10.1371/journal.pntd.0001044 (PMC3091835; doi:10.1371/journal.pntd.0001044)
Supplement: Protocol S1 — Trial Protocol: downloaded from http://clinicaltrials.gov/ct2/show/NCT00765024. (0.06 MB DOC) [file pntd.0001044.s002.doc]

**Trial Protocol: downloaded from** **http://clinicaltrials.gov/ct2/show/NCT00765024**

**Ivermectin Versus Albendazole for Chronic Strongyloidiasis**

**This study is enrolling participants by invitation only.**

First Received: September 30, 2008   Last Updated: June 18,

| **Sponsor:** | Mahidol University |
| --- | --- |
| **Collaborator:** | Atlantic Laboratory Ltd |
| **Information provided by:** | Mahidol University |
| **ClinicalTrials.gov Identifier:** | NCT00765024 |


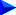
**Purpose**

A prospective controlled trial to compare the efficacy and safety of 7-day albendazole, single dose ivermectin, and 2-single dose ivermectin in 72 patients with chronic strongyloidiasis will be conducted at Siriraj Hospital, Bangkok, Thailand.

| [**Condition**](http://clinicaltrials.gov/ct2/help/conditions_desc) | [**Intervention**](http://clinicaltrials.gov/ct2/help/interventions_desc) | [**Phase**](http://clinicaltrials.gov/ct2/help/phase_desc) |
| --- | --- | --- |
| Chronic Strongyloidiasis | Drug: Ivermectin Drug: ivermectin Drug: Albendazole | Phase III |

| Study Type: | Interventional |
| --- | --- |
| Study Design: | Allocation: Randomized Control: Active Control Endpoint Classification: Safety/Efficacy Study Intervention Model: Parallel Assignment Masking: Open Label Primary Purpose: Treatment |
| Official Title: | Efficacy and Safety of Ivermectin Versus Albendazole for Chronic Strongyloidiasis |

**Resource links provided by NLM:**

[Drug Information](http://druginfo.nlm.nih.gov/drugportal/drugportal.jsp) available for: [Albendazole](http://clinicaltrials.gov/ct2/bye/jQoPWw4lZXcPSi7iedN6ZXNxvdDxuQ7Ju6c9cXcPSi7iEd-yWB7EZ6o35Q1yzB-VuQUgEscxkd789V7ap67x5B1eZdh8.) [Ivermectin](http://clinicaltrials.gov/ct2/bye/4QoPWw4lZXcPSi7iedN6ZXNxvdDxuQ7Ju6c9cXcPSi7iEd-yWB7EZ6o35Q1yzB-VuQUgEscxkd789D16zwNLzB-PedJ.)

[U.S. FDA Resources](http://clinicaltrials.gov/ct2/info/fdalinks)

**Further study details as provided by Mahidol University:**

Primary Outcome Measures:

- cure rate [ Time Frame: 1 year ] [ Designated as safety issue: No ]

Secondary Outcome Measures:

- safety [ Time Frame: 1 year ] [ Designated as safety issue: Yes ]

| Estimated Enrollment: | 72 |
| --- | --- |
| Study Start Date: | July 2008 |
| Estimated Study Completion Date: | June 2010 |
| Estimated Primary Completion Date: | June 2010 (Final data collection date for primary outcome measure) |

| [**Arms**](http://clinicaltrials.gov/ct2/help/arm_group_desc) | [**Assigned Interventions**](http://clinicaltrials.gov/ct2/help/interventions_desc) |
| --- | --- |
| Albendazole: Active Comparator  Albendazole for 7 days | Drug: Albendazole  Albendazole 7 days |
| ivermectin: Experimental  ivermectin 200 mcg/kg single dose | Drug: Ivermectin  single dose of 200 mcg/kg |
| ivermectin 2 doses: Experimental  ivermectin 200 mcg/kg two doses in 2 weeks | Drug: ivermectin  two single dose of 200mcg/kg in 2 weeks |


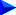
**Eligibility**

| Ages Eligible for Study: | 18 Years to 90 Years |
| --- | --- |
| Genders Eligible for Study: | Both |
| Accepts Healthy Volunteers: | No |

**Criteria**

Inclusion Criteria:

- Patients with positive strongyloides larva in the stool

Exclusion Criteria:

- Pregnancy
- Lactating women
- Known allergy to any study drug


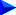
**Contacts and Locations**

Please refer to this study by its ClinicalTrials.gov identifier: NCT00765024

Locations

| **Thailand, Bangkok** | |
| --- | --- |
| Siriraj Hospital |  |
| Bangkoknoi, Bangkok, Thailand, 10700 | |

**Sponsors and Collaborators**

Mahidol University

Atlantic Laboratory Ltd

Investigators

| Principal Investigator: | Yupin Suputtamongkol, MD | Faculty of Medicine, Siriraj Hospital, Mahidol University |
| --- | --- | --- |


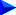
**More Information**

No publications provided

| Responsible Party: | Faculty of Medicine Siriraj Hospital ( Mahidol University ) |
| --- | --- |
| ClinicalTrials.gov Identifier: | [NCT00765024](http://clinicaltrials.gov/ct2/show/NCT00765024)     [History of Changes](http://clinicaltrials.gov/ct2/archive/NCT00765024) |
| Other Study ID Numbers: | TM001-2008 |
| Study First Received: | September 30, 2008 |
| Last Updated: | June 18, 2010 |
| Health Authority: | Thailand: Ethical Committee |

Keywords provided by Mahidol University:

| strongyloidiasis, ivermectin, albendazole |
| --- |

Additional relevant MeSH terms:

| Strongyloidiasis Rhabditida Infections Secernentea Infections Nematode Infections Helminthiasis Parasitic Diseases Albendazole Ivermectin Anticestodal Agents Antiplatyhelmintic Agents Anthelmintics | Antiparasitic Agents Anti-Infective Agents Therapeutic Uses Pharmacologic Actions Antiprotozoal Agents Tubulin Modulators Antimitotic Agents Mitosis Modulators Molecular Mechanisms of Pharmacological Action Antineoplastic Agents |
| --- | --- |
